# Three-Day Continuous Exposure Monitoring of CNT Manufacturing Workplaces

**DOI:** 10.1155/2015/237140

**Published:** 2015-06-01

**Authors:** Ji Hyun Lee, Kang Ho Ahn, Sun Man Kim, Ellen Kim, Gun Ho Lee, Jeong Hee Han, Il Je Yu

**Affiliations:** ^1^Institute of Nanoproduct Safety Research, Hoseo University, Asan 336-795, Republic of Korea; ^2^Hanyang University, Ansan, Gyeonggi-do 426- 791, Republic of Korea; ^3^Occupational Safety & Health Research Institute, Korean Occupational Safety & Health Agency, Daejeon 305-380, Republic of Korea

## Abstract

Continuous monitoring for possible exposure to carbon nanotubes was conducted over a period of 2 to 3 days at workplaces that manufacture multiwall carbon nanotubes (MWCNTs) and single wall carbon nanotubes (SWCNTs). To estimate the potential emission of carbon nanotubes (CNTs) and potential exposure of workers, personal sampling, area monitoring, and real-time monitoring using an scanning mobility particle sizer (SMPS) and dust monitor were conducted at workplaces where the workers manufactured CNTs. The personal and area sampling of the total suspended particulate (TSP) at the MWCNT manufacturing facilities ranged from 0.031 to 0.254 and from N.D (not detected) to 0.253 mg/m^3^, respectively. This 2- to 3-day monitoring study found that nanoparticles were released when opening the chemical vapor deposit (CVD) reactor door after the synthesis of MWCNTs, when transferring the MWCNTs to containers and during blending and grinding. However, distinguishing the background concentration from the work process particle emission was complicated due to sustained and even increased particle concentrations after the work processes were terminated. The MWCNTs sampled for transmission electron microscopy (TEM) observation exhibited a tangled shape with no individual dispersed CNT structures.

## 1. Introduction 

With the commercialization of nanotechnology, exposure usually starts from the workplace and then spreads to environment and consumer exposure. Exposure assessment is an important element for understanding the potential risks of nanomaterials, as such results can be used to identify emission sources and evaluate the performance of a control approach, compliance with exposure limits, and risk estimation.

While the safety sponsorship program of the OECD WPMN (working party on manufactured nanomaterials) provides some information on the physicochemical properties, toxicity, and ecotoxicity of 13 representative nanomaterials, exposure data on nanomaterials remains limited. Thus, recognizing the importance of exposure data for both risk assessment and risk characterization and management, the OECD recently asked the member countries to participate in exposure assessment case studies related to worker, consumer, and environmental exposure.

However, there is no current consensus on the best sampling method for characterizing exposure to CNTs. Comparing particle concentrations at the emission source with background particle concentrations has been frequently used to identify emission sources of nanomaterials qualitatively and implement measures for exposure mitigation [1–4]. Yet, various approaches can be applied to characterize exposure in a particular environment.

Accordingly, this study was conducted to provide exposure information on CNT manufacturing workplaces based on three days of continuous exposure monitoring. The number-based particle size distributions were determined using an SMPS and dust monitor, while filter-based sampling was used to monitor the mass concentrations of suspended particles in the air. As a result, particle exposure information was obtained to estimate the conditions at CNT manufacturing workplaces and protect the workers from exposure.

## 2. Materials and Methods

### 2.1. Sampling Sites

The current study measured the nanoparticle concentrations inside two plants manufacturing MWCNTs and SWCNTs in 2011. The information related to each plant is shown in Table 1.

### 2.2. Personal and Area Sampling

The air samples were taken by drawing air through mixed cellulose ester filters in sampling cassettes (37 mm diameter, 0.8 *μ*m nominal pore-size, and 2 in. cowl, open-face) obtained from Pall Corp (P/N 64678, Michigan USA). The filter samples for the personal sampling were collected in the breathing zone using MSA (Escort Elf pump) operated sampling pumps at a flow rate of 1.5–2.0 L/min and SKC (Leland Legacy pump) operated sampling pumps at a flow rate of 6.9–7.3 L/min when the work duration was short. Two sampling holders were also changed during the sampling period to avoid overload (the holders are marked as “ ^*∗*^” in Table 2 and the data expressed as TWA concentration).The sampling with personal samplers was performed during the normal work period from 09:30 to 16:00 and typically lasted from 159 to 350 min. The personal samplers were attached to workers involved in manufacturing nanomaterials. Area samples were also collected by placing the samplers 1–4 meters away from the manufacturing devices, at suspected emission sources of nanoparticles, and at several representative locations to represent the workplace.

### 2.3. Real-Time Aerosol Monitoring

An SMPS combining a differential mobility analyzer (DMA, 4220, HCT Co., Ltd., Korea) and condensation particle counter (CPC, 4312, HCT Co., Ltd., 0–10^8^ particles/cm^3^ detection range) was used to monitor the particle size distribution with an electrical mobility diameter ranging from 15 to 710.5 nm. Meanwhile, a dust monitor (Model 1.109, Grimm) was used to observe the particle size distribution with a diameter ranging from 0.25 to 32 *μ*m. The workplace air was sampled at a low rate of 0.3 and 1.2 L/min for the SMPS and dust monitor, respectively. The SMPS scanned the particle sizes at a time resolution of 2.5 min (120 s for up-scan and 30 s for retrace), while the average time for the dust monitor was 1 min. The real-time aerosol monitoring lasted 3 days at the MWCNT manufacturing workplace and 2 days at the SWCNT manufacturing workplace.

### 2.4. Transmission Electron Microscopy (TEM)

The air samples were analyzed according to National Institute of Occupational Safety and Health (NIOSH) analytical method 7402 [5] and Han et al. [6]. The filters were coated with carbon and mounted on carbon-coated copper grids (Veco, Eerbeek, Holland) using acetone vapor. Plus, the CNT and Ag nanoparticles were morphologically identified using a scanning transmission electron microscope (STEM; Hitachi 7100, Tokyo) and determined by comparing the elemental composition of the CNT and Ag nanoparticles using an energy dispersive X-ray analyzer (EDX; KEVEX 7000Q, Foster City, CA) [2, 6].

## 3. Results

### 3.1. MWCNT Manufacturing Workplace (Workplace A)

**Figure 1:**
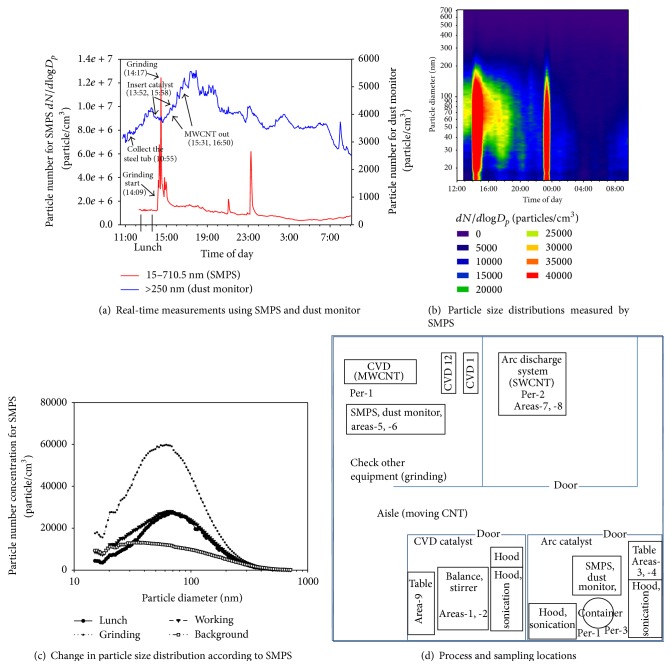
Real-time particle measurements at workplace A (MWCNT production, 1st day).

Workplace A was a large-scale MWCNT manufacturing workplace that used a thermal CVD (chemical vapor deposition) process, as shown in Figure 1(d). Workplace A also manufactured SWCNTs using an arc discharge process. As shown in Figure 1(d), workplace A had a CVD synthesis room, CVD catalyst room, and arc catalyst room. While the CVD room was an open space, the CVD catalyst room and arc catalyst room were both closed spaces. The MWCNTs manufactured in the CVD synthesis room were produced 5 times/day at 210-minute intervals, with a maximum production of 10 times/day. Plus, 100 g of MWCNTs were usually produced using 5 g of catalysts. The MWCNTs were transferred to a container in the arc catalyst room and ground using a mixer for use with an arc stick. The measurements were taken in front of the CVD equipment on the first day, although equipment problems resulted in continuous grinding on the first day. As the arc catalyst room was a small-size room and included frequent MWCNT transfers, a relatively high exposure was predicted. Thus, measurements were also taken in the arc catalyst room on the second and third day (time course of events at workplace A was described in supplement 1 in Supplementary Material available online at http://dx.doi.org/10.1155/2015/237140). The process and measurement locations are shown in Figure 1(d). The mass concentrations of the total suspended particulate (TSP) were also measured at the same locations and showed some changes during the 3-day measurement (Table 2). After their work periods, the workers usually removed dust from their bodies using an air gun, which also removed some of the black color on the filters as the sampling cassettes were open face. Thus, the mass concentration of personal exposure may have been underestimated due to the removal of particles by the air gun. The personal and area TSP-TWA (8 hr) concentrations ranged from 0.031 to 0.254 and N.D to 0.174 mg/m^3^, respectively, the arithmetic mean concentrations for the personal and area sampling were 0.111 and 0.067 mg/m^3^, respectively, and the geometric mean concentrations for the personal and area sampling were 0.092 and 0.053 mg/m^3^, respectively (Table 3). On the first day, personal sampling was taken in front of SWCNT synthesis equipment, but the sampling could not be conducted the second and third day due to SWCNT synthesis equipment failure. Area samplings were, however, taken from the first day to third day to study changes of TSP concentration in the workplace. The concentrations were 0.13421, 0.02137, and N.D for the first day, second day, and third day, respectively (Table 2).

#### 3.1.1. Measurements Taken in front of CVD Equipment CVD (1st Day)

On the 1st day, measurements were taken in front of the CVD equipment, yet equipment problems necessitated continuous grinding and cleaning after lunch (12:30–13:30, supplement 1), which resulted in increased particle numbers measured by both the SMPS and the dust monitor (Figures 1(a) and 1(c)). The grinding significantly increased the number of concentrations when considering the size distribution (Figure 1(b)), as particles under 200 nm increased during the normal operation and grinding, with a particular increase in 60–70 nm particles (Figure 1(c)). These particle numbers were sustained even after the termination of work at 18:00, indicating that the particles generated during the equipment operation continuously influenced the particle concentration (Figure 1(a)). *dN*/*d*log*D*_*p*_ ranged from 361,975 to 12,445,239 particles/cm^3^ according to the SMPS and the particle number ranged from 2,508 to 5,599 particles/cm^3^ according to the dust monitor.

#### 3.1.2. MWCNT Handling (2nd and 3rd Day)

**Figure 2:**
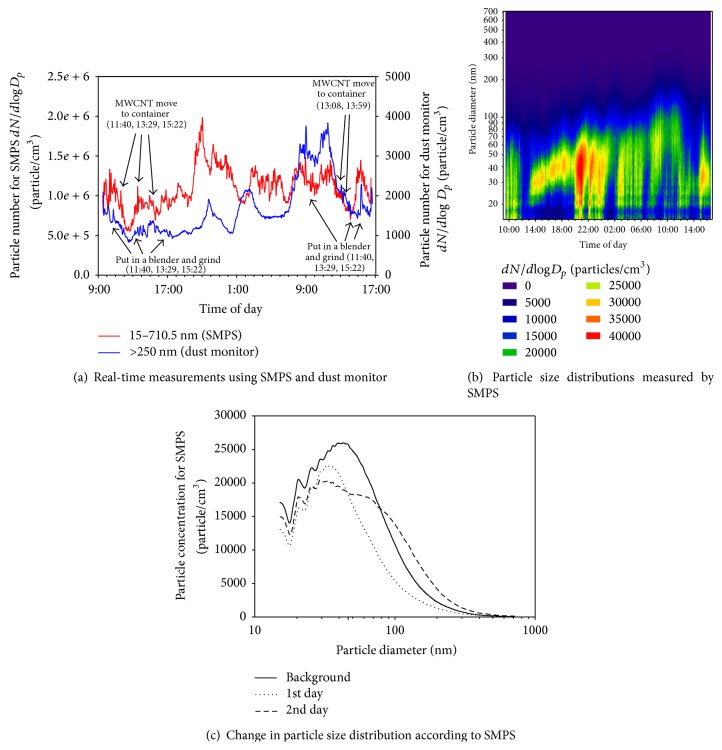
Real-time particle measurements at workplace A (MWCNT handling, 2nd and 3rd day).

**Figure 3:**
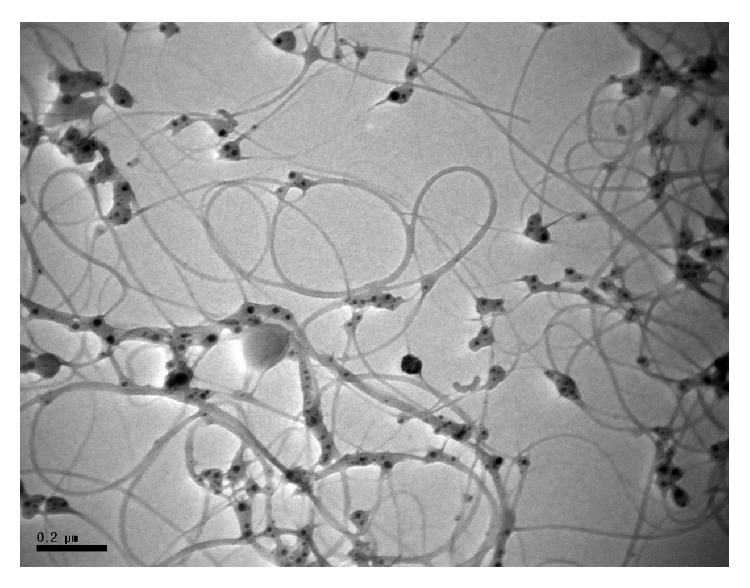
TEM micrograph of sample right after opening CVD.

On the 2nd and 3rd day, the arc stick manufacturing operation using MWCNTs was measured in the arc catalyst room. The arc catalyst room was 39.6 m^2^ and had a push-pull door. The MWCNTs were transferred to a large container, and then certain amounts were taken out and ground using a mixer to make arc sticks throughout the day. The particle number was higher after 18:00 than during the working hours (Figures 2(a) and 2(b)). Plus, the background particle number concentration was higher than the concentration measured during the working hours, with more particles under 100 nm and a 30–50 nm maximum (Figure 2(c)). This result suggests that particles generated during the manufacturing operation remained even after the termination of the operation. Moreover, the increase of particles larger than 250 nm on the 3rd day indicated the continuous presence of CNTs in the work atmosphere (Figure 2(a)), with particle numbers ranging from 550,819 to 1,972,775 particles/cm^3^ (*dN*/*d*log*D*_*p*_) according to the SMPS and from 831 to 3,842 particles/cm^3^ according to the dust monitor.  Figure 3 shows the TEM morphology of the MWCNTs right after starting to operate the CVD equipment, where the MWNCTs contain catalysts (black dots) and have a tangled shape.

### 3.2. SWCNT Manufacturing Workplace (Workplace B)

**Figure 4:**
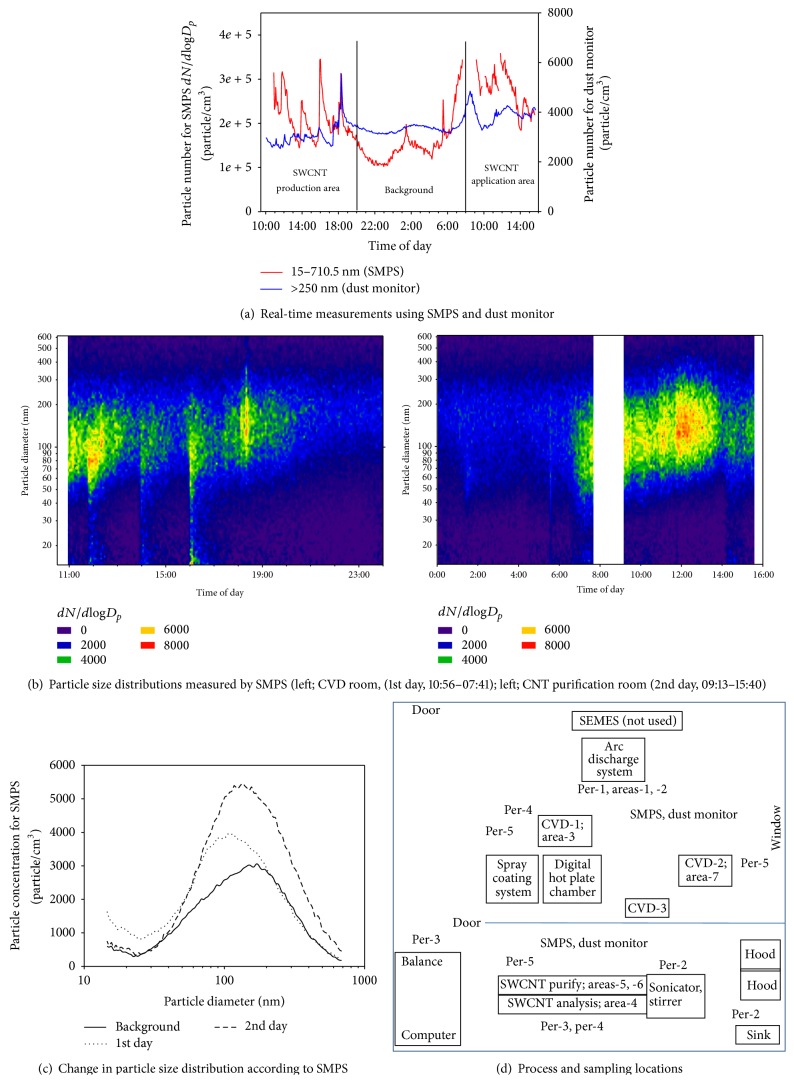
Real-time particle measurements at workplace B.

Workplace B manufactured SWCNTs using an arc discharge method. The work space included a variety of manufacturing equipment and a separate handling area for sonication, stirring, and weighting. The door between the manufacturing and handling areas remained open all the time, and workers moved frequently between the two areas. The work space and measurement points are shown in Figure 4(d). Time course of events at workplace B was describe in supplement 2. For the personal and area sampling, the TSP-TWA (8 hr) concentrations ranged from 0.102 to 0.277 and 0.042 to 0.223 mg/m^3^, respectively, the arithmetic mean concentrations were 0.168 and 0.144 mg/m^3^, respectively, and the geometric mean concentrations were 0.161 and 0.129 mg/m^3^, respectively (Table 5).

#### 3.2.1. First Day

The SWCNTs were manufactured and collected once from the arc discharge system on the 1st day. The operation mainly involved removing impurities from the SWCNTs using CVD, where the SWNCTs were placed in the CVD equipment for a while, the CVD door was then opened to allow the CNTs to be stirred using a stick, the door was then closed and the CVD area vacuumed 1-2 times, and finally the SWCNTs were collected. Black particles were observed outside the CVD equipment. For the personal and area sampling TSP concentration ranged from 0.10165–0.16364 mg/m^3^ and 0.06136–0.21307 mg/m^3^, respectively (Table 4). The particle numbers ranged from 101,621 to 345,800 particles/cm^3^ (*dN*/*d*log*D*_*p*_) according to the SMPS and from 2,523 to 5,552 particles/cm^3^ according to the dust monitor (Figure 4(a)). The particle sizes were distributed between 60 and 300 nm (Figure 4(b)).

#### 3.2.2. Second Day

Measurements were taken in the handling room where the manufactured SWCNTs were pretreated. For the personal and area sampling TSP concentration ranged from 0.14789 to 0.27662 mg/m^3^ and from 0.04217 to 0.22316 mg/m^3^, respectively (Table 4). The particle numbers ranged from 183,885 to 358,912 particles/cm^3^ (*dN*/*d*log*D*_*p*_) according to the SMPS and from 3,266 to 4,273 particles/cm^3^ according to the dust monitor. The particle sizes were similarly distributed as on the 1st day between 60 and 300 nm. However, the particle size distribution shifted to larger than 100 nm on the 2nd day when compared with the 1st day (Figure 4(c)). The background particle number concentrations were measured after 18:00 on the 1st day and before 08:00 on the 2nd day and were clearly lower on the 1st and 2nd day according to the SMPS, yet they were not lower on the 1st day according to the dust monitor (Figure 4(c)). One explanation is that particles generated during the work period on the 1st day remained in the work atmosphere even after the work was terminated and increased in size from the sizes detected by the SMPS to the sizes detected by the dust monitor.

## 4. Discussion

The current authors already investigated MWCNT exposure at several MWCNT manufacturing facilities [2] and identified various release work processes, such as opening the CVD door, spraying, CNT preparation, ultrasonic dispersion wafer heating, and opening the water bath cover. However, the investigations at seven MWCNT manufacturing facilities were mostly conducted on a single day, which did not allow thorough characterization of the CNT release and exposure and also made it difficult to compare the background particle concentration with the work process particle concentration. Furthermore, these one-day studies were unable to characterize the nanoparticle release pattern and influence of outdoor particle penetration during and after the work period. Therefore, the current exposure monitoring study was conducted at MWCNT and SWCNT manufacturing facilities over 2-3 days. While some release processes, such as opening the CVD door, transferring the MWCNTs to containers, blending, and grinding, released nanoparticles during the work period, such number of concentrations were sustained and even increased after the work period was terminated.

This study was conducted before the recommended CNT occupational exposure limits were announced by the US NIOSH. After reviewing animal and other toxicological data relevant to assessing the potential nonmalignant adverse respiratory effects of CNTs and CNFs, the NIOSH recommended 1 *μ*g/m^3^ elemental carbon (EC) as a respirable mass 8 h TWA (time-weighted average) for CNTs and CNFs. Although this recommended exposure limit (REL) is expected to reduce the risk of pulmonary inflammation and fibrosis, there is still some residual risk at the REL and uncertainty concerning chronic health effects, including whether some types of CNT may be carcinogenic; thus continued efforts should be made to reduce exposure as much as possible [7]. Based on the NIOSH REL, the OSHA then published a fact sheet on working safely with nanomaterials in 2013 which recommended that worker exposure to respirable carbon nanotubes and carbon nanofibers should also not exceed 1 *μ*g/m^3^ as an 8 h TWA, [8]. While the current study only reported the TSP mass concentrations at the CNT workplaces, which do not represent the CNT concentrations, the TSP mass concentrations can still indicate the degree of exposure to CNTs in the workplace. From the current CNT exposure assessment done by Lee et al. [9], percentage of EC from TSP can be estimated. About 4% of TSP were estimated for EC from their exposure data and a respirable fraction of EC was 25%, as suggested by Erdely et al. [10]. Therefore, 1% of TSP could be estimated for respirable EC. Workers could be exposed to 0.3–2.5 *μ*g/m^3^ of EC in the workplace A and 1–2.8 *μ*g/m^3^ of EC in the workplace B, indicating being exposed slightly over the NIOSH REL 1 *μ*g/m^3^.

Several attempts have recently been made to distinguish the background concentration from CNT exposure. Yet the use of various catalytic metals in CNT synthesis makes it very complicated to distinguish the background from CNT exposure [9, 11]. The current attempt to distinguish the background from CNT exposure was also unsuccessful due to increased particle numbers after the work period was terminated. This increase may have been due to outdoor particles penetrating inside the facility or sustained particle concentrations even after the work processes were terminated.

This study also attempted to count the CNT tube structures in transmission electron microscopy grid openings, as described in the previous study by Han et al. [6]. Yet, most of the TEM micrographs obtained from MWCNT manufacturing process in this study did not show the dispersed individual tube structures seen in the study by Han et al. Instead, all the MWNCT structures were tangled structures containing catalyst metals, and the dispersed CNT structures were process-dependent, as suggested by Lee et al. [2].

The exposure assessment of carbon nanotubes, such as SWCNTs and MWCNTs, remains a challenge in the field of industrial hygiene, as there have been relatively few CNT sampling and monitoring studies, and the optimal sampling filters and methods have not yet been established. Direct reading instruments for particle counting and size distribution, such as a CPC and OPC, are unable to represent CNTs exactly, while size measurements using DMAS (or SMPS) do not always work due to the arc charge caused by the charged CNTs in the DMA [12]. Although several attempts have been made to count the CNT structures using TEM and other microscopic methods [6, 13, 14], there are still no standard methods for CNT counting. In addition, determining the mass concentration of CNTs based on measuring the elemental carbon remains a challenge due to the detection limits and complicated nature of current analytical methods. Yet, despite these difficulties in assessing exposure to nanomaterials and CNTs, guidelines and papers have been published to guide and harmonize strategies for exposure measurement [15, 16].

## Supplementary Material

Time course of events during 3-day continuous exposure monitoring of CNT manufacturing workplaces at Workplace A (Supplement 1) and Workplace B (supplement 2) is described in the Supplement.

## Figures and Tables

**Table 1:** Information on workplaces.

Plant	Region (handling workers)	Manufactured materials	Process	Engineering controls	PPE Used
A (Industry)	Incheon (5)	MWCNT manufacturing, application	Chemical synthesis (pilot test)	Enclosed local exhaust system, fume hood	Half-mask, working clothes, gloves

B (Industry)	Jeonju (6)	SWCNT manufacturing, application	Chemical synthesis (mass production)	Enclosed local exhaust system	Working clothes

**Table 2:** TSP mass concentrations (8 hr TWA) in personal and area samples from workplace A.

Process	Sampling site	Sampling day	Filter weight (Before)	Filter weight (After)	Flow rate (L/min)	Sampling time (min)	Mass concentration (mg/m^3^)	8 hr TWA (mg/m^3^)
MWCNT synthesis	Personal-1	1st	0.04524	0.04535	2.001	308	0.17848	0.11453
		2nd	0.04469	0.04493	1.972	375	0.32454	0.25355
		3rd	0.03856	0.03867	1.972	370	0.15076	0.11621

SWCNT synthesis	Personal-2	1st	0.04503	0.04506	1.995	244	0.06163	0.03133

ARC Catalyst	Personal-3	1st	0.04496	0.04501	2.046	317	0.07709	0.05091
		2nd	0.0447	0.04482	1.985	336	0.17992	0.12594
		3rd	0.03905	0.0392	1.985	359	0.34831	0.25189^*∗*^
	Personal-4	2nd	0.04468	0.04479	2.042	356	0.15132	0.11223
		3rd	0.03895	0.03899	2.042	163	0.12018	0.04081

CVD Catalyst	Area-1	1st	0.04482	0.04493	2.020	293	0.18585	0.11345
		2nd	0.03861	0.03865	2.001	370	0.05403	0.04165
		3rd	0.03873	0.03877	1.950	317	0.06471	0.04274
	Area-2	1st	0.04478	0.04492	7.027	284	0.07016	0.04151
		2nd	0.0449	0.04508	6.773	370	0.07183	0.05537
		3rd	0.03879	0.03886	6.773	87.2	0.11852	0.02153
	Area-9	2nd	0.03894	0.03903	2.010	438	0.10223	0.09328
		3rd	0.03883	0.039	2.026	375	0.22376	0.17481

ARC Catalyst	Area-3	1st	0.04473	0.04484	1.953	172	0.32746	0.11734
		2nd	0.04493	0.04507	1.960	380	0.32109	0.24447^*∗*^
		3rd	0.03852	0.03859	2.001	370	0.09455	0.07288
	Area-4	1st	0.04513	0.04524	6.833	292	0.05513	0.03354
		2nd	0.0447	0.04486	7.033	249	0.09137	0.04740
		3rd	0.03893	0.03898	7.010	367	0.01944	0.01486

In front of MWCNT equip.	Area-5	1st	0.04513	0.04519	2.007	298	0.10035	0.06230
		2nd	0.04454	0.04465	2.026	212	0.25610	0.11311
		3rd	0.03901	0.03903	1.960	364	0.02803	0.02126
	Area-6	1st	0.0444	0.04456	6.848	298	0.07841	0.04868
		2nd	0.0448	0.04497	7.088	318	0.07542	0.04997
		3rd	0.03868	0.03873	7.033	132	0.05386	0.01481

In front of SWCNT equip.	Area-7	1st	0.04468	0.04481	2.018	159	0.40516	0.13421
		2nd	0.03864	0.03866	1.950	363	0.02825	0.02137
		3rd	0.03867	0.0373	2.010	131	ND	ND
	Area-8	1st	0.04464	0.04484	7.328	265	0.10299	0.05686
		2nd	0.04452	0.04475	7.010	365	0.08989	0.06835

ND: Not detected; ^*∗*^changed during the sampling period to avoid overload.

**Table 3:** TSP concentrations (8 hr TWA) at workplace A.

	Range (mg/m^3^)	Mean (mg/m^3^)	SD (mg/m^3^)	GM (mg/m^3^)	GSD
Personal sampling	0.031–0.254	0.122	0.082	0.097	2.10
Area sampling	N.D–0.244	0.072	0.057	0.055	2.15

*Note*. Mean: arithmetic mean; SD: standard deviation; GM: geometric mean; GSD: geometric standard deviation.

**Table 4:** TSP mass concentrations (8 hr TWA) in personal and area samples from workplace B (2nd day).

Process	Sampling site	Manufacturing area (1st)						Handling area (2nd)					
		Filter weight (before)	Filter weight (after)	Flow rate (L/min)	Sampling time (min)	Mass concentration (mg/m^3^)	TWA (mg/m^3^)	Filter weight (before)	Filter weight (after)	Flow rate (L/min)	Sampling time (min)	Mass concentration (mg/m^3^)	TWA (mg/m^3^)
Arc system	Personal-1	0.04514	0.04527	1.994	398	0.16385	0.13586	0.04489	0.04507	2.053	380	0.23073	0.18266

CNT analysis	Personal-2	0.0449	0.04506	2.037	312	0.25175	0.16364	0.04500	0.04522	2.038	297	0.36355	0.22495
	Personal-3	0.04502	0.04514	2.078	311	0.18573	0.12034	0.04488	0.04515	2.034	261	0.50872	0.27662
	Personal-4	0.04492	0.04502	2.050	322	0.15153	0.10165	0.04507	0.04522	2.113	299	0.23742	0.14789

CNT purify	Personal-5	0.04494	0.04509	2.024	171	0.43340	0.15440	0.04492	0.04509	2.086	277	0.29428	0.16982

Arc system	Area-1	0.04487	0.04503	1.946	181	0.45425	0.17129	0.04486	0.04502	2.016	381	0.20836	0.16538
	Area-2	0.04496	0.04509	4.414	169	0.17427	0.06136	0.04498	0.04523	4.462	303	0.18491	0.11673

CVD-1	Area-3	0.04486	0.04499	1.919	181	0.37427	0.14113	0.04516	0.04530	1.973	307	0.23119	0.14787

CVD-2	Area-7	NM						0.04484	0.04504	1.948	307	0.33451	0.21395

CNT analysis	Area-4	0.04493	0.04513	1.956	320	0.31961	0.21307	0.04500	0.04504	1.976	306	0.06615	0.04217

CNT purify	Area-5	0.04498	0.04516	2.017	319	0.27982	0.18597	0.04495	0.04516	1.961	304	0.35235	0.22316
	Area-6	0.04492	0.0451	4.409	292	0.13983	0.08506	0.04492	0.04515	4.566	299	0.16847	0.10494

NM: not measured.

**Table 5:** TSP concentrations (8 hr TWA) at workplace B.

	Range (mg/m^3^)	Mean (mg/m^3^)	SD (mg/m^3^)	GM (mg/m^3^)	GSD
Personal sampling	0.102–0.277	0.168	0.051	0.161	1.34
Area sampling	0.042–0.223	0.144	0.059	0.129	1.67

*Note*. Mean: arithmetic mean; SD: standard deviation; GM: geometric mean; GSD: geometric standard deviation.
