# Supplementary material for: Three-Day Continuous Exposure Monitoring of CNT Manufacturing Workplaces
Source: Biomed Res Int. 2015 Jun 1;2015:237140. doi: 10.1155/2015/237140 (PMC4466344; doi:10.1155/2015/237140)
Supplement: Supplementary file 1 — Time course of events during 3-day continuous exposure monitoring of CNT manufacturing workplaces at Workplace A (Supplement 1) and Workplace B (supplement 2) is described in the Supplement. [file 237140.f1.docx]

Supplement 1. Time course of events at workplace A.

| **Day** | **Time** | **Operation** | **Temp/**  **Humidity** |
| --- | --- | --- | --- |
| 1st day | 10:53 | CVD door opened for collection of MWCNTs and then tightly sealed |  |
|  | 10:55 | MWCNTs transferred to ARC catalyst room and tightly sealed |  |
|  | 11:18 | Start of SMPS measurements |  |
|  | 11:36 | Workers leave workplace for lunch | 19%, 16.2℃ |
|  | 12:30-13:30 | Lunch time | 19%, 17℃ |
|  | 13:52 ~ 13:58 | Door open for insertion of catalysts | 18%, 17.6℃ |
|  | 14:09 | Grinding to repair troubled equipment (14:17 ~ 14:18, 14:27 ~ 14:28, 14:35 ~ 14:36, 14:48 ~ 14:49, 14:55 ~ 14:56, 15:26, and so on) |  |
|  | 14:12 ~ 14:13 | Removal of grinding debris | 18%, 17.9℃ |
|  | 15:20 | Cleaning |  |
|  | 15:31 | CVD door opened and manufactured MWCNTs transferred to arc catalyst room |  |
|  | 15:33 | Cleaning of CVD area |  |
|  | 15:35 | MWCNTs poured into large container(vacuum cleaning) |  |
|  | 15:56 | Catalysts moved in front of CVD |  |
|  | 15:58 | Catalysts placed in CVD(397℃) |  |
|  | 16:00 | Door closed (381℃ program started) |  |
|  | 16:13 ~ 16:31 | Door of SEMES equipment opened several times to draw new design. |  |
|  | 16:50 | Door opened and manufactured MWCNTs transferred to arc catalyst room(735℃) |  |
|  | 16:51 | MWCNTs collected and area cleaned with vacuum |  |
| 2nd day | 10:00 ~ 10:05 | Measurements started (during manufacture of arc sticks in fume hood) | 22%, 11.9℃ |
|  | 10:08 ~ 10:13 | Arc sticks manufactured in fume hoods |  |
|  | 10:30 ~ 10:37 | Arc sticks manufactured in left fume hood |  |
|  | 10:33 ~ 10:50 | MWNCTs transferred from right fume hood to left fume hood, ground finely using blender, and transferred to product container |  |
|  | 10:41 | Arc sticks manufactured in left fume hood |  |
|  | 10:50 ~ 10:56 | Vacuum cleaning |  |
|  | 11:40 ~ 11:44 | MWCNTs transferred to large container |  |
|  | 11:44 | Vacuum cleaning |  |
|  | 11:48 | Catalysts prepared in CVD catalyst room |  |
|  | 11:50 ~ 13:00 | Lunch | 20%, 12.5℃ |
|  | 13:29 ~ 13:35 | MWCNTs collected from SEMES and transferred to large container (dust found at bottom of container and in corridor |  |
|  | 13:35 ~ 13:43 | MWCNTs removed using vacuum | 20%, 13℃ |
|  | 14:02 ~ 14:05 | Arc sticks manufactured in left fume hood |  |
|  | 14:26 ~ 14:27 | MWNCTs transferred from right fume hood to left fume hood, ground finely using blender, and transferred to product container |  |
|  | 14:26 ~14:43 | MWCNTs weighed from various containers and dust generated in left fume hood |  |
|  | 14:44 ~ 15:02 | MWNCTs transferred from right fume hood to left fume hood, ground finely using blender, and transferred to product container |  |
|  | 14:45 ~ 14:53 | MWCNTs weighed from various containers and dust generated in left fume hood, arc sticks manufactured |  |
|  | 15:02 ~15:03 | Vacuum cleaning(twice) |  |
|  | 15:22 ~ 15:37 | MWCNTS collected from SEMES and transferred to large container, dusting off, and vacuum cleaning |  |
|  | 16:26 ~ 16:57 | MWNCTs transferred from right fume hood to left fume hood, ground finely using blender, and transferred to product container | 19%, 14.4℃ |
|  | 16:56 | Cleaning |  |
|  | 16:42 | MWNCTs transferred from right fume hood to left fume hood, ground finely using blender, and transferred to product container |  |
|  | 16:54 | Cleaning |  |
|  | 16:57 ~ 17:01 | Vacuum cleaning and catalysts prepared in CVD |  |
| 3rd day | 9:43 | Catalysts prepared in CVD |  |
|  | 9:57 ~ 11:16 | MWNCTs transferred from right fume hood to left fume hood, ground finely using blender, and transferred to product container |  |
|  | 10:00 ~ 10:14 | Vacuum cleaning of left fume hood |  |
|  | 10:00 ~ 10:14 | MWCNTs ground using blender and distributed in left hood |  |
|  | 10:26 ~ 10:53 | MWCNTs ground using blender and distributed in left hood, arc sticks manufactured |  |
|  | 10:29 ~ 10:45 | MWCNTs ground using blender, distributed in left hood, and transferred to product container, plus occasional vacuum cleaning |  |
|  | 11:20 ~ 11:29 | MWCNTs transferred to small product container |  |
|  | 11:29 ~ 11:45 | MWCNTs ground using blender, distributed in left hood, and weighed in various containers, plus vacuum cleaning | 23%, 12.9℃ |
|  | 11:50 ~ 13:00 | Lunch |  |
|  | 12:40 | MWCNTs collected from SEMES and transferred to large container in catalyst room |  |
|  | 13:08 ~ 13:23 | MWNCTs transferred from right fume hood to left fume hood, plus occasional vacuuming |  |
|  | 13:08 ~ 13:27 | Arc sticks manufactured in left hood |  |
|  | 13:16 ~ 13:23 | Cleaning |  |
|  | 13:21 ~ 13:23 | Cleaning (twice) |  |
|  | 13:59 ~ 14:10 | MWCNTs collected from SEMES and transferred to large container in catalyst room |  |
|  | 14:00 ~ 14:10 | Frequent vacuum cleaning during transfer |  |
|  | 14:10 ~ 14:39 | MWNCTs transferred from right fume hood to left fume hood, ground finely using blender, and transferred to product container. Plus occasional vacuum cleaning at 1-2-minute intervals |  |
|  | 15:18 ~ 15:21 | Arc sticks manufactured in left hood. |  |
|  | 15:25 ~ 16:17 | MWNCTs transferred from right fume hood to left fume hood, ground finely using blender, and transferred to product container, plus occasional vacuum cleaning at 1-2-minute intervals |  |
|  | 16:17 ~ 16:21 | Operation terminated, vacuum cleaning, and leaving workplace after shower |  |

Supplement 2. Time course of events at workplace B.

| Day | Time | Operation | Temp/  Humidity |
| --- | --- | --- | --- |
| 1st day | 10:56 | Measurements started |  |
|  | 11:17 | CVD-1 opened and closed |  |
|  | 11:25-26 | CVD-2 opened, vacuum cleaning of CVD floor and entrance, and CVD door closed. |  |
|  | 11:34 | CVD-1 opened |  |
|  | 11:40-48 | CVD-1 opened and tilted to brush off SWCNTs.  CVD-1 replaced and CNTs stirred with stick to mix evenly. Door entrance cleaned with tissue paper, CNTs stirred one more time with stick, CVD door closed, and gas injected into CVD-1. Vacuum cleaning |  |
|  | 11:54 | CVD-1 opened, CNTs stirred with stick, and CVD door closed. |  |
|  | 12:05-1:20 | Lunch |  |
|  | 13:32-56 | Arc discharger opened, CNTs collected and brushed-off, plus vacuum cleaning | 25%, 21.8℃ |
|  |  | CNTs collected in bottle, vacuum cleaning, and new container placed in arc discharger. |  |
|  | 13:48-50 | CVD-1 opened and SWCNTs brushed off. Vacuum cleaning |  |
|  | 13:51-54 | CNTs stirred with stick to mix evenly, CVD door closed, and vacuum cleaning |  |
|  | 14:06 | CVD-1 opened, CNTs stirred with stick to mix evenly, and CVD door closed |  |
|  | 14:22-26 | CVD-2 opened, CNTs stirred with stick to mix evenly, and CVD door closed. Vacuum cleaning |  |
|  | 14:32, 14:55 | CVD-1 opened, CNTs stirred with stick to mix evenly, and CVD door closed |  |
|  | 14:56 | CVD-2 opened, CNTs stirred with stick to mix evenly, and CVD door closed |  |
|  | 15:19 | SMPS and dust monitor placed in between CVD-1 and CVD-2. |  |
|  | 15:53-57 | CVD-1 opened, CNTs stirred with stick to mix evenly, and CVD door closed. Vacuum cleaning |  |
|  | 16:15-20 | CVD-2 opened, CNTs stirred with stick to mix evenly, and CVD door closed. Vacuum cleaning |  |
|  | 16:21 | CVD-1 opened, CNTs stirred with stick to mix evenly, and CVD door closed. |  |
| 2nd day | 9:09 | Measurements started | 27%, 19℃ |
|  | 9:26-29, 9:33-38, 10:22-42 | CNTs weighed, ground using blender, weighed again, and transferred to container. Vacuum cleaning |  |
|  | 11:11-44 | CNTs weighed, ground using blender, weighed again, and transferred to container repeatedly |  |
|  | 12:00-13:00 | Lunch |  |
|  | 13:18-19 | Blending operation | 29%, 20.1℃ |
|  | 13:23-24 | CNTs weighed, ground using blender, weighed again, and transferred to container |  |
|  | 13:25-29 | CNTs collected in small bottle. |  |
|  | 13:30-35 | CNTs collected in large bottle, separated, and cut. Vacuum cleaning |  |
|  | 13:41-44 | CNTs weighed, ground using blender, weighed again, and transferred to small bottle |  |
|  | 13:48-50 | CNTs weighed. Vacuum cleaning. |  |
|  | 13:56-14:02 | CNTs weighed, ground using blender, weighed again, and transferred to container repeatedly. Vacuum cleaning |  |
|  | 14:20-24, 14:27-30, 14:31-43, 15:04-07, 15:11-14 | CNTs separated | 29% 20.3℃ |
|  | 14:25, 14:29 | CNTs weighed, ground using blender, weighed again, and transferred to container |  |
|  | 14:54-56 | CNTs weighed and vacuum cleaning |  |
|  | 15:22-23 | CNTs separated and cut finely using scissors |  |
|  | 15:37 | Termination of measurements |  |
